# Relationships between serum Klotho concentrations and cognitive performance among older chronic kidney disease patients with albuminuria in NHANES 2011-2014

**DOI:** 10.3389/fendo.2023.1215977

**Published:** 2023-07-25

**Authors:** Jialing Zhang, Aihua Zhang

**Affiliations:** ^1^ Department of Nephrology, Xuanwu Hospital, Capital Medical University, Beijing, China; ^2^ The National Clinical Research Center for Geriatric Disease, Xuanwu Hospital, Capital Medical University, Beijing, China

**Keywords:** albuminuria, chronic kidney disease, cognition impairment, Klotho, NHANES

## Abstract

**Background:**

The potential relationship between Klotho and cognitive function is limited and controversial. This study aimed to quantify the association of Klotho and cognitive impairment in chronic kidney disease (CKD) patients with albuminuria.

**Methods:**

Serum Klotho was measured by enzyme-linked immunosorbent assay. Patients with urine albumin to creatinine ratio (UACR) > 30mg/g from the National Health and Nutrition Survey (NHANES) 2011-2014 were divided into 4 groups according to the quartile of Klotho. Cognitive function was examined using the Consortium to Establish a Registry for Alzheimer’s Disease (CERAD), Digit Symbol Substitution Test (DSST), and Animal Fluency Test. The relationship between Klotho and cognitive function was analyzed by multivariable regression and subgroup analysis.

**Results:**

Among 368 CKD patients with albuminuria, we found that Klotho was negatively associated with creatinine, and positively associated with hemoglobin, and estimated glomerular filtration rate. No significant linear relationship was showed between Klotho (as a continuous variable) and cognitive function. When regarded Klotho as a category variable, patients in the quartile 3 group were at a better cognitive performance for CEARD-word learning subset and DSST, especially in the CKD patients with 30 mg/g < UACR <300 mg/g, but not in participants with UACR > 300 mg/g.

**Conclusions:**

The increased Klotho was associated with an increased cognitive function in CKD patients with microalbuminuria. Further studies are needed to demonstrate whether Klotho may be a beneficial biomarker of cognitive health and neurodegeneration.

## 1 Introduction

Chronic kidney disease (CKD) is defined as abnormal kidney structures and function that affects health and lasts for at least 3 months, characterized by the decrease in glomerular filtration rate (GFR) and the increase in urinary albumin (1). The presence of albuminuria was defined as urine albumin-to-creatinine ratio (UACR) ≥ 30 mg/g. Evidence has reported associations between an increased risk of cognitive impairment and CKD (2, 3).

Three forms of Klotho protein have been identified, including full-length transmembrane Klotho (m-Klotho), soluble Klotho (s-Klotho), and secreted Klotho (4). mKlotho is a single-pass transmembrane protein, and can be shed into sKlotho or secreted Klotho by proteases or alternative mRNA splicing. Klotho has pathophysiological association with various disease, including malignancies, vascular calcification, cardiovascular disease, and renal fibrosis (5–8). Various studies have demonstrated that systemic Klotho levels are downregulated in CKD, indicating a risk for CKD progression (9, 10).

Klotho is detected mainly in distal convoluted tubules of the kidney and choroid plexus in the brain (11), in addition, it is also expressed in the cerebral cortex and cerebellum (12). In a study including subjects aging 39 to 83, α-Klotho in the cerebrospinal fluid may be an important biomarker of neurodegeneration (13). The cerebrospinal fluid Klotho concentrations are lower in Alzheimer’s disease (14), probably leading to a poor synaptic and cognitive functions (15). Besides, the relationship between cognitive functions and Klotho was also suggested in schizophrenia (16), Alzheimer’s disease (17, 18), and multiple system atrophy (19). In humans, a genetic variant of Klotho gene is independently associated with enhanced cognition (20). However, the prognostic value of Klotho for declining of cognition in CKD patients with proteinuria was still under debate.

Therefore, the purpose of this study was to evaluate the association between serum Klotho concentrations and cognitive dysfunction in CKD patients with different level of albuminuria.

## 2 Methods

### 2.1 Study population

**Figure 1:**
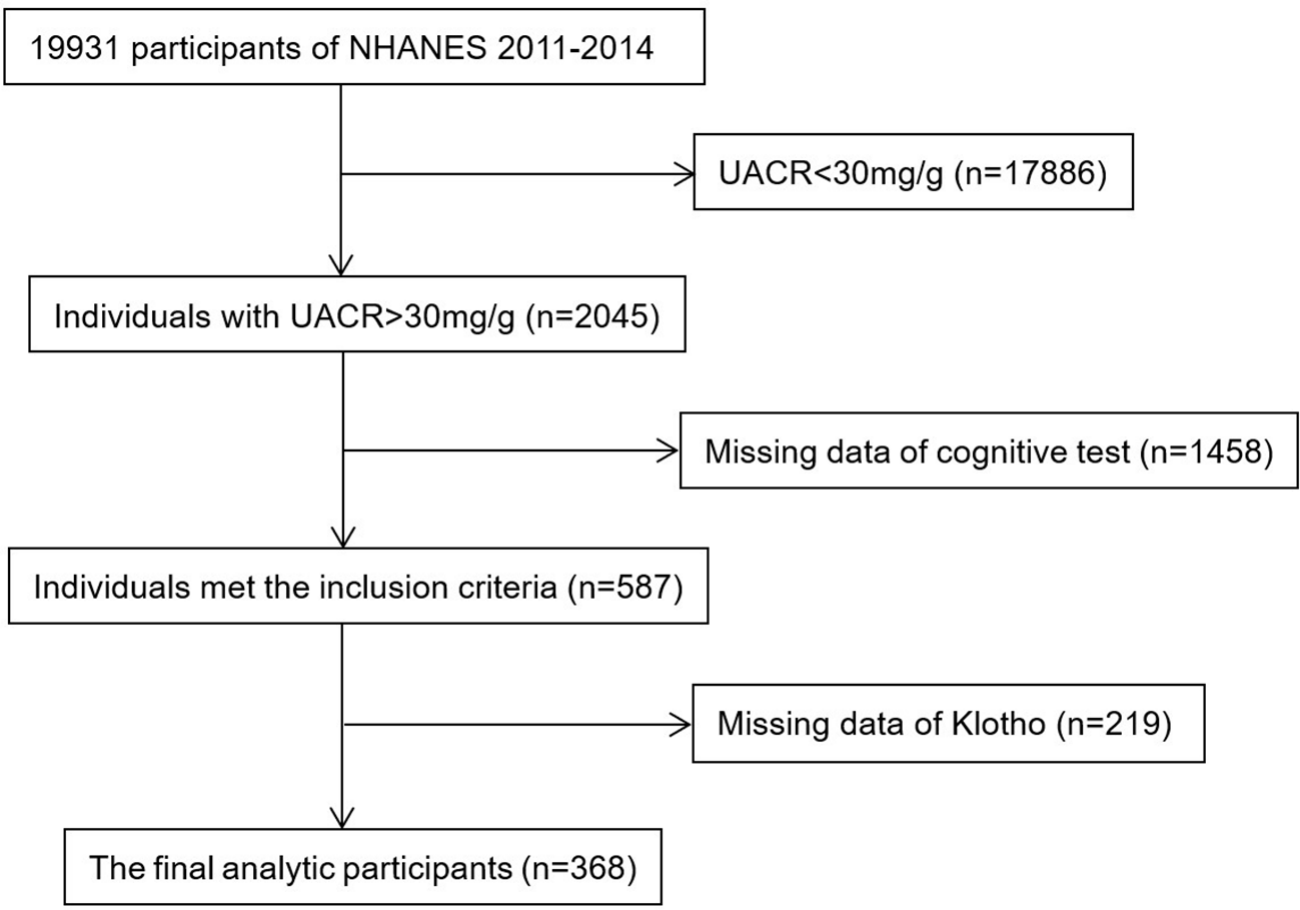
Flow chart of patients’ enrollment.

NHANES was conducted biannually in the United States to provide a representative sample of the civilian, non-institutionalized U.S. population (https://www.cdc.gov/nchs/nhanes/about_nhanes.htm). Cognitive Function Tests were measured in two cycles of NHANES (2011–2012 and 2013–2014) for the older population. During this inclusion screening process, 2045 participants with UACR > 30mg/g were available for analysis. We excluded 1458 and 219 participants when missing data of cognitive tests and serum Klotho concentration, respectively. Finally, there were 368 participants in our analysis, and the detailed flow chart was shown in **Figure 1**. The CKD stages were defined with eGFR and/or evidence of kidney damage according to the KDIGO guidelines: stage 1, eGFR ≧ 90 mL/min/1.73 m2 with UACR ≥ 30 mg/g; stage 2, eGFR 60–89 mL/min/1.73 m2 with ACR ≥ 30 mg/g; stage 3, eGFR 30–59 mL/min/1.73 m2; stage 4, eGFR 15–29 mL/min/1.73 m2; stage 5, eGFR < 15 mL/min/1.73 m2 (21). NHANES was performed by the Centers for Disease Control and Prevention (CDC), the National Center for Health Statistics (NCHS) Ethics Review Board authorized the study, and all participants gave their informed permission.

### 2.2 Cognitive performance test

NHANES performed a series of assessments for cognitive performance among participants aged 60 years or older, including Consortium to Establish a Registry for Alzheimer’s Disease Word List Learning Test (CERAD-WL), the CERAD Word List Recall Test (CERAD-DR), the Animal Fluency test (AFT) and the Digit Symbol Substitution Test (DSST).

CERAD is designed to assess immediate and delayed learning of new verbal information in the memory sub-domain (22). CERAD consisted of three consecutive learning trials and a delayed recall. The AFT examined categorical verbal fluency, a component of executive function, which was applied to distinguish between persons who have normal cognitive functioning and those who have mild cognitive impairment or more severe forms of cognitive impairment (23). Participants were given a minute to name as many animals as they could in a minute. DSST relied on the integrity of executive function, processing speed, attention, spatial perception, and visual scanning (24). Participants had two minutes to match the symbols corresponding to numbers.

### 2.3 Measurement of serum soluble Klotho

All samples were stored at -80°C until they were analyzed. Klotho concentrations were analyzed by a commercially available enzyme-linked immunosorbent assay (ELISA) kit produced by IBL International, Japan. The details about laboratory methodology as well as the quality assurance and monitoring are discussed by NHANES.

### 2.4 Covariates

Covariates, such as age, sex (male and female), educational level (less than high school, high school, and higher than high school), smoking status, drinking status, and questionnaire findings (self-reported physician diagnosis of hypertension, and diabetes), laboratory data (white blood cell, hemoglobin, albumin, blood urea nitrogen (BUN), creatinine, calcium, phosphate, alkaline phosphatase (ALP), cholesterol and triglyceride) were gathered by uniform interviews, physical and laboratory testing, and questionnaires administered by medical personnel.

### 2.5 Statistical analysis

All data were analyzed by SPSS statistics 23. To describe normal variables, we used mean ± standard deviation (SD), and the median (interquartile range) was for non-normally distributed variables. Categoric variables were described by percentage. Patients were divided into four groups according to the level of Klotho. Kruskal–Wallis test was used to analyze the difference between continuous variables, while Chi-square test was used between classified variables. Serum Klotho concentrations were logarithmic transformed due to its skew distribution. Pearson correlation analysis was performed to explore the association between various covariates and Klotho. We further used multivariable linear regression models to examine the relationship between each cognitive test score and Klotho. The relationship between log-transformed (Klotho) and each cognitive scores was evaluated by restricted cubic spline models fitted for linear regression models with 3 knots at the 10th, 50th, and 90th percentiles. The level of significance was defined as P < 0.05.

## 3 Results

### 3.1 Population characteristics

**Table 1** showed the characteristics of the patients divided by quartile of serum Klotho levels. The prevalence of diabetes, hypertension, smoking status, drinking status, and the level of hemoglobin, albumin, BUN, creatinine, eGFR, calcium and phosphate were significantly different among four groups. In addition, the value of serum Klotho were different among the four groups and the quartile 2 group had the lowest cognitive value.

**Table 1:** Characteristics of the included participants (stratified by quartile of Klotho).

	Total (n=368)	Quartile 1 (n=92)	Quartile 2 (n=92)	Quartile 3 (n=92)	Quartile 4 (n=92)	P
Age (year)	68 (63-73)	68.5 (64-74)	69 (64-74)	67 (63.25-71)	67 (62-72)	0.128
Male (%)	51.8	43.5	55.4	58.7	49.5	0.497
Race (%)						
Mexican American (%)	4.4	6.3	3.6	5.4	3.6	0.733
Other Hispanic (%)	6	1.8	9.9	8.1	9.1	0.1
Non-Hispanic White (%)	50.9	51.4	45	41.4	35.5	0.315
Non-Hispanic Black (%)	33.5	35.1	34.2	34.2	48.2	0.277
Other (%)	5.2	5.4	7.2	10.8	3.6	0.198
Education						
Less than 9th grade (%)	16.1	14.1	17.4	14.1	18.7	0.834
9−11th grade (%)	18.5	19.6	27.2	7.6	19.8	0.021
High school graduate (%)	24.5	23.9	22.8	27.2	24.2	0.94
College or AA degree (%)	24.5	26.1	18.5	30.4	23.1	0.409
College graduate or above (%)	16.4	16.3	14.1	20.7	14.3	0.642
Diabetes (%)	44.7	42.4	50	38	48.4	0.042
Hypertension (%)	74.4	81.5	70.7	72.8	72.5	<0.001
Smoke (%)	36.5	32.6	33.7	40.2	39.6	<0.001
Drink (%)	62.9	60.9	57.6	73.9	59.3	<0.001
White blood cell (10^9/l)	7.1 (6.1-8.6)	7.35 (6.13-9.08)	7.15 (5.95-8.75)	7.2 (6.23-8.68)	7.1 (5.7-8.1)	0.388
Hemoglobin (g/dl)	13.6 ± 1.52	13.22 ± 1.57	13.42 ± 1.51	14.05 ± 1.47	13.71 ± 1.43	0.002
Platelet (10^9/l)	211 (182-258)	220.5 (184.5-262.75)	215.5 (183-263)	211 (174.25-258.75)	203 (186-246)	0.245
Albumin (g/l)	42 (39-44)	41.5 (39-44)	41 (38-43)	42 (40-44)	41 (40-43)	0.026
BUN (mg/dl)	11 (5.71-16)	11 (6.87-17.96)	12 (7.85-17.75)	10.36 (5.36-16)	10 (4.64-16)	0.021
Creatinine (μmol/l)	88.4 (71.6-114.92)	95.47 (75.8-120)	95.03 (71.82-144.98)	79.56 (68.29-99.01)	85.75 (65.42-110.5)	0.002
eGFR (ml/min/1.73 m^2^)	70.17 ± 26.95	63.26 ± 26.66	65.47 ± 30.68	78.98 ± 22.74	73.03 ± 24.51	<0.001
UACR (mg/g)	69.02 (41.71-175.83)	94.14 (43.87-261.39)	80.89 (43.18-197.41)	61.23 (41.2-167.14)	59.47 (38-114.49)	0.071
Cholesterol (mmol/l)	4.71 (3.88-5.66)	4.9 (3.9-5.61)	4.54 (3.78-5.66)	4.78 (4.18-5.81)	4.55 (3.85-5.69)	0.479
Triglyceride (mmol/l)	1.63 (1.07-2.47)	1.5 (0.98-2.21)	1.55 (1.03-2.6)	1.78 (1.08-2.85)	1.77 (1.12-2.41)	0.418
Calcium (mmol/l)	2.35 (2.3-2.43)	2.38 (2.33-2.43)	2.33 (2.3-2.38)	2.38 (2.3-2.43)	2.35 (2.3-2.45)	0.006
Phosphate (mmol/l)	1.2 (1.2-1.32)	1.26 (1.13-1.38)	1.15 (1.04-1.3)	1.16 (1.07-1.32)	1.2 (1.07-1.29)	0.02
Alkaline phosphatase (mmol/l)	72 (56-88)	72 (56-89.75)	67 (54-82.75)	71.5 (55.75-87.5)	77 (65-95)	0.052
CERAD-WL score	19 (14-21)	19 (14-21)	17 (12.25-20.75)	19 (16-22)	19 (15-22)	0.04
CERAD-DR score	6 (4-7)	6 (4-7)	6 (4-7)	6 (4-8)	6 (4-7)	0.187
Animal frequency test score	15 (11-19)	15.58 ± 6.5	14 (11-18)	16 (12-21)	15 (11-18)	0.04
DSST score	38.37 ± 19.7	37.88 ± 19.92	35.25 ± 18.22	42.98 ± 19.76	37.37 ± 20.34	0.06
Klotho (pg/ml)	781.8 (611.8-982.3)	513.85 (456.38-570.13)	706.3 (659.88-750.65)	851.3 (807.63-901.93)	1206.8 (1081.9-1331.4)	<0.001

AF, Animal Fluency test; CERAD-WL, Consortium to Establish a Registry for Alzheimer’s Disease Word Learning test; CERAD-DR, Consortium to Establish a Registry for Alzheimer’s Disease Delayed Recall test; DSST, Digit Symbol Substitution test; CKD, chronic kidney disease; BUN, blood urea nitrogen; eGFR, estimated glomerular filtration rate; UACR, urea albumin-creatinine ratio.

Among 1458 participants without cognition tests, 340 participants are aged 60 years and above. The characteristics of the older participants without cognition tests are summarized in **Supplementary file Table 1**. Compared with the included participants, the excluded participants were more likely to be older, other race/ethnicity, and had poorer residual renal function.

### 3.2 Correlated factors for Klotho

As shown in **Table 2**, positive association between hemoglobin, while negative association between creatinine and Klotho was observed. However, in our Pearson correlation analysis, we did not find any significant relationship between each cognitive test and serum Klotho (shown as a continuous variable).

**Table 2:** Correlation between Klotho and related factors.

	R	P
Age	-0.094	0.072
Hemoglobin	0.126	0.016
Albumin	0.009	0.865
BUN	-0.095	0.07
Creatinine	-0.136	0.009
eGFR	0.136	0.009
UACR	-0.068	0.194
Cholesterol	0.002	0.966
Triglyceride	0.092	0.079
Calcium	0.057	0.28
Phosphate	-0.099	0.059
Alkaline phosphatase	0.068	0.197
CERAD-WL	0.066	0.209
CERAD-DR	0.058	0.268
Animal frequency test	-0.035	0.507
DSST	-0.002	0.963

AF, Animal Fluency test; CERAD-WL, Consortium to Establish a Registry for Alzheimer’s Disease Word Learning test; CERAD-DR, Consortium to Establish a Registry for Alzheimer’s Disease Delayed Recall test; DSST, Digit Symbol Substitution test; BUN, blood urea nitrogen; eGFR, estimated glomerular filtration rate; UACR, urea albumin-creatinine ratio.

### 3.3 Klotho concentrations and performance on cognitive tests

**Figure 2:**
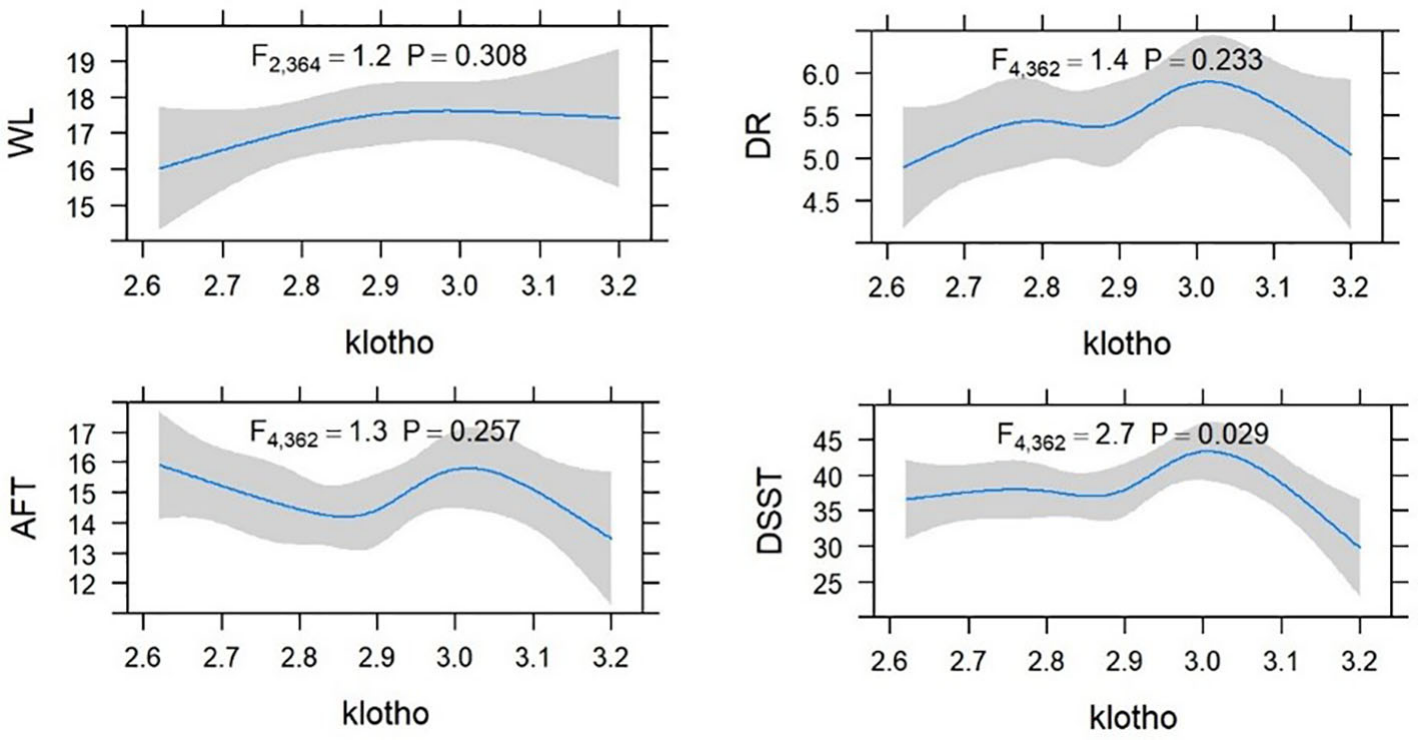
Restricted cubic spline (RCS) plot of the association between Klotho and cognitive function in CKD.

Higher level of serum Klotho were significantly associated with lower odds of cognitive impairment in patients with UACR > 30mg/g. Comparing to the quartile 2 group, patients in the quartile 3 group had higher scores of CERAD-WL and DSST. However, patients with a higher quartile of Klotho were not with a better cognitive function assessed by AFT. The associations between cognitive function test scores and Klotho are shown in **Table 3**, and there was no significant association between each cognitive test and serum Klotho regarded as a continuous variable. A non-linear association was found between Klotho and DSST score (shown in **Figure 2**).

**Table 3:** Multiple linear regression analysis for each cognitive test and Klotho.

	CERAD-WL		CERAD-DR		AFT		DSST	
	Standardization coefficient β (95%CI)	P	Standardization coefficient β (95%CI)	P	Standardization coefficient β (95%CI)	P	Standardization coefficient β (95%CI)	P
Klotho (continuous variable)	0.076 (-1.059,6.397)	0.16	0.062 (-0.652,2.521)	0.248	-0,07 (-6.407,1.626)	0.188	-0.026 (-15.084,8.927)	0.614
Quartile 1	0.067 (-0.804,2.477)	0.317	0.003 (-0.684,0.717)	0.964	0.139 (0.135,3.504)	0.034	0.049 (-3.479,7.717)	0.457
Quartile 2	Reference		Reference		Reference		Reference	
Quartile 3	0.143 (0.158,3.434)	0.032	0.09 (-0.217,1.182)	0.175	0.107 (-0.287,3.077)	0.104	0.136 (0.298,11.338)	0.039
Quartile 4	0.107 (-0.302,2.989)	0.109	0.025 (-0.567,0.839)	0.704	0.031 (-1.286,2.095)	0.638	0.038 (-3.979,7.203)	0.571

Adjust for age, sex, diabetes, hypertension, smoke, drink, hemoglobin, albumin, BUN, creatinine, calcium, phosphate, alkaline phosphatase, cholesterol, triglyceride.

AF, Animal Fluency test; CERAD-WL, Consortium to Establish a Registry for Alzheimer’s Disease Word Learning test; CERAD-DR, Consortium to Establish a Registry for Alzheimer’s Disease Delayed Recall test; DSST, Digit Symbol Substitution test; OR, odds ratio; CI, confidence interval.

### 3.4 Serum Klotho and cognitive performance across different level of albuminuria

Subgroup analyses were performed to demonstrate how the relations between Klotho and cognitive performance varied across different level of UACR. In patients with UACR ranging from 30 to 300mg/g, only under the CERAD-WL test, patients in the quartile 3 group of serum Klotho had a better cognitive test score comparing to quartile 2. At the same time, there was nonsignificant difference between each cognitive test and Klotho levels in patients with UACR > 300mg/g (shown in **Table 4**).

**Table 4:** Subgroup analysis for each cognitive test and Klotho based on albuminuria.

	CERAD-WL		CERAD-DR		AFT		DSST	
	Standardization coefficient β (95%CI)	P	Standardization coefficient β (95%CI)	P	Standardization coefficient β (95%CI)	P	Standardization coefficient β (95%CI)	P
UACR > 300mg/g								
Klotho (continuous variable)	-0.022 (-13.701,11.888)	0.887	0.084 (-3.508,6.132)	0.587	-0.058 (-13.367,8.818)	0.682	0.07 (-25.981,43.872)	0.609
Quartile 1	0.077 (-3.89,5.872)	0.685	0.061 (-1.492,2.08)	0.742	0.062 (-3.285,4.802)	0.707	0.022 (-11.494,20.254)	0.894
Quartile 2	Reference		Reference		Reference		Reference	
Quartile 3	0.159 (-2.992,7.456)	0.394	0.317 (-0.229,3.594)	0.083	0.298 (-0.369,8.287)	0.072	0.195 (-5.673,22.627)	0.234
Quartile 4	-0.015 (-6.099.5.622)	0.935	-0.02 (-2.266,2.023)	0.91	-0.109 (-6.485,3.225)	0.503	0.09 (-11.494,20.254)	0.581
30mg/g UACR < 300mg/g								
Klotho (continuous variable)	0.096 (-0.722,7.262)	0.108	0.055 (-0.927,2.576)	0.355	-0.083 (-7.192,1.176)	0.158	-0.048 (-19.066,7.73)	0.406
Quartile 1	0.023 (-1.566,2.151)	0.757	-0.033 (-0.996,0.638)	0.667	0.134 (-0.175,3.72)	0.074	0.025 (-5.168,7.33)	0.734
Quartile 2	Reference		Reference		Reference		Reference	
Quartile 3	0.151 (0.042,3.627)	0.045	0.064 (-0.445,1.132)	0.392	0.083 (-0.812,2.945)	0.265	0.06 (-3.507,8.549)	0.411
Quartile 4	0.09 (-0.702,2.855)	0.234	-0.011 (-0.842,0.723)	0.881	0.011 (-1.727,2.001)	0.885	-0.036 (-7.479,4.484)	0.622

Adjust for age, sex, diabetes, hypertension, smoke, hemoglobin, albumin, BUN, creatinine, calcium, phosphate, alkaline phosphatase, cholesterol, triglyceride.

AF, Animal Fluency test; CERAD-WL, Consortium to Establish a Registry for Alzheimer’s Disease Word Learning test; CERAD-DR, Consortium to Establish a Registry for Alzheimer’s Disease Delayed Recall test; DSST, Digit Symbol Substitution test; UACR, urea albumin-creatinine ratio; OR, odds ratio; CI, confidence interval.

## 4 Discussion

In the current study, the relationship between serum Klotho and cognitive dysfunction in CKD patients with albuminuria was analyzed. We found that a relatively higher level of Klotho was significantly associated with a better cognition function. However, in CKD patients with UACR > 300mg/g, the association of Klotho and cognition function was slight. Our results probably presented a non-linear association of Klotho and cognitive impairment in CKD.

CKD is a critical health burden worldwide. Both low eGFR and albuminuria are both independent risk factors for cognitive impairment (25, 26). Cognitive impairment is common in end-stage renal disease patients (27, 28). The pathophysiology of CKD-related cognitive impairment was multifactorial, including vascular disease, cerebrovascular disease, the toxicity of uremic toxins, depression, sleep disturbance, anemia, and so on (2). However, the relationship and pathophysiological mechanism of Klotho on poor cognitive function has not been fully elucidated.

The Klotho gene exists in three paralogs: αKlotho (referred to as Klotho here), βKlotho, and γKlotho (29). The extracellular domain of mKlotho can be shed constitutively by proteases and yields sKlotho (30). Increasing evidence showed that a close association between Klotho and kidney diseases (31, 32). Besides, the relationship between Klotho and cognitive functions has been explored in general older adults (33) and various diseases, containing schizophrenia (16, 34), Alzheimer’s disease (17) and cerebrovascular diseases (35). Klotho is probably a potential key player in the metabolic coupling between neurons and astrocytes (36). A growing number of studies also examined the association of Klotho with adverse outcomes (37–39). However, whether Klotho in CKD patients could affect cognitive function is still inconclusive.

In our study, we collected and analyzed relevant data from NHANES 2011-2014. As a result, we found patients with albuminuria in the quartile 3 group obtained better results in cognition-related tests, when comparing to the quartile 2 group. Our finding also showed that the serum Klotho concentration was negatively correlated with age and creatinine, and positively associated with eGFR, which was consistent with previous findings (40, 41). Although an inverse relationship between serum α-Klotho and proteinuria was shown in CKD patients (42), the association was not significant in our study.

The mechanism underlying low serum Klotho levels increasing the risk of cognitive impairment is multifactorial. Klotho was reported to protect against fibrosis, apoptosis, and autophagy (43–45). Reduced circulating Klotho levels are also associated with enhanced oxidative stress and inflammation (46). Upregulated Klotho levels could inhibit oxidative stress and inflammation by suppressing insulin/IGF-1 signaling and nuclear factor-κB (47, 48). Cognitive dysfunction associated with Klotho has been demonstrated before (49, 50). Serum and cerebrospinal fluid protein levels of α-Klotho were positively correlated, and both of them were strongly correlated with cognition scores (13). Additionally, circulating Klotho concentration is even higher in cerebrospinal fluid than in serum regardless of KL-VS genotype (51). The variation in Klotho gene is associated with bigger brain volume and better cognitive function in older adults (52). In CKD rats, the expression of α-Klotho significantly declined in the hippocampus, which might affect the cognitive function (53). Furthermore, elevating Klotho in mice enhanced synaptic plasticity, enriched synaptic receptor subunit for learning and memory, and intrinsic connectivity in fronto-parietal (20, 54). In another nephrectomized induced cognitive impairment rodent model, Klotho levels decreased in frontal cortex but not in hippocampus, accompanying with an increase in NF-κB and tumor necrosis factor-alpha levels (55).

Interestingly, in our subgroup analysis according to the different level of UACR, we only found a significant relationship between higher Klotho level and better cognitive performance on CERAD-WL test in patients with microalbuminuria (30mg/g < UACR < 300mg/g), but not in patients with UACR > 300mg/g. A probable non-linear association of Klotho and cognitive dysfunction was also presented in this article. In a study including patients with diabetic kidney disease, a positive correlation was found between Klotho and reduction of eGFR, and the tubular injury marker (56). Other previous studies documented significantly higher levels of Klotho with CKD (57) or even nonsignificant relationship between residual renal function and serum Klotho (40, 58). Klotho is also produced by extra-renal organs (59). Previous study revealed a key role for local vascular Klotho as an endogenous inhibitor of vascular calcification, demonstrating the vascular territory as another source of serum soluble Klotho (60). The opposite finding probably suggested that a reduction of tubular excretion of sKlotho due to a severe proximal tubule injury, which might lead to an increasing in serum Klotho concentrations. A heavy proteinuria might reflect an aggravated renal tubule damage and poor renal function. In our study, we found that the association of Klotho and cognitive function was not significant in patients with UACR > 300mg/g. Patients with macroalbuminuria probably had a higher risk of other comorbidities, hospitalization and mortality (61, 62). Our results infer that the accumulating Klotho due to a severe kidney damage could not benefit for a better cognitive function. Although the interaction of albuminuria and Klotho on cognition is inconclusive, an early detection and comprehensive evaluation of Klotho in different stage of CKD is essential. More research is warranted to confirm the predictive value of Klotho on cognition for older CKD patients.

Several limitations should still be considered. Although several covariates were adjusted to remove potential confounding factors, we could not exclude some other residual confounding. Our study did not include enough information of other comorbidities. Moreover, this is an observational study, the association of Klotho and cognition could not be interpreted as causality. Additionally, we only explore the association of serum Klotho and cognition by ELISA, the level of Klotho protein in tissues is unknown and unpredicted. The diagnostic sensitivity and specificity of Klotho ELISA kit could be affected by the freshness of serum samples. Finally, our results may not be expanded to the participants under the age of 60. The excluded patients without cognition scores may have ethnic, and renal function differences, leading to select bias. Further research is needed to elucidate the explore potential mechanisms of Klotho and cognition function in patients with different age and stage of CKD.

## 5 Conclusion

Overall, our results suggest that a relatively higher level of serum Klotho may positively correlate with a better cognitive performance in CKD patients with albuminuria. Our study proposed that the detection of Klotho should be taken into consideration in the early stage of kidney damage, and clinicians should incorporate Klotho detection into routine assessments for older CKD patients aiming at cognitive enhncement.

## Data availability statement

The original contributions presented in the study are included in the article/**Supplementary Material**. Further inquiries can be directed to the corresponding author.

## Ethics statement

The studies involving human participants were reviewed and approved by The study was approved by the NCHS Research Ethics Review Board (Continuation of Protocol #2011–17). The patients/participants provided their written informed consent to participate in this study.

## Author contributions

AZ contributed to the study concept and design. JZ contributed to data collection. JZ, and AZ contributed to the statistical analysis. JZ contributed to the original draft. AZ contributed to the review draft. All authors contributed to the article and approved the submitted version.
