# Supplementary material for: Relationships between serum Klotho concentrations and cognitive performance among older chronic kidney disease patients with albuminuria in NHANES 2011-2014
Source: Front Endocrinol (Lausanne). 2023 Jul 25;14:1215977. doi: 10.3389/fendo.2023.1215977 (PMC10407554; doi:10.3389/fendo.2023.1215977)
Supplement: Supplementary file 1 [file Table_1.docx]

**Supplemental Table 1** Baseline characteristics between the participants aged ≥ 60 years with and without cognition tests

|  | Participants with cognition tests (n=368) | Participants without cognition tests (n=340) | P |
| --- | --- | --- | --- |
| Age (year) | 68 (63-73) | 80 (71-80) | <0.001 |
| Male (%) | 51.8 | 52.1 | 0.31 |
| Race (%) |  |  |  |
| Mexican American (%) | 4.4 | 15.4 | <0.001 |
| Other Hispanic (%) | 6 | 10.3 | <0.001 |
| Non-Hispanic White (%) | 50.9 | 31.2 | <0.001 |
| Non-Hispanic Black (%) | 33.5 | 28.7 | <0.001 |
| Other (%) | 5.2 | 14.3 | <0.001 |
| Education |  |  |  |
| Less than 9th grade (%) | 16.1 | 21.5 | 0.223 |
| 9−11th grade (%) | 18.5 | 16.2 | 0.241 |
| High school graduate (%) | 24.5 | 23.5 | 0.443 |
| College or AA degree (%) | 24.5 | 21.5 | 0.183 |
| College graduate or above (%) | 16.4 | 16.8 | 0.853 |
| Diabetes (%) | 44.7 | 39.1 | 0.134 |
| Hypertension (%) | 74.4 | 77.3 | 0.366 |
| Hemoglobin (g/dl) | 13.6 (12.6-14.7) | 13.2 (12.1-14.2) | <0.001 |
| Albumin (g/l) | 42 (39-44) | 41 (39-43) | 0.477 |
| BUN (mg/dl) | 11 (5.71-16) | 18 (13-25) | <0.001 |
| Creatinine (μmol/l) | 88.4 (71.6-114.92) | 96.36 (73.37-128.18) | 0.145 |
| UACR (mg/g) | 69.02 (41.71-175.83) | 86.27 (47.51-194.36) | 0.263 |
| eGFR (ml/min/1.73m^2^) | 70.12 (53.32-86.95) | 58.46 (43.64-75.51) | <0.001 |
| Cholesterol (mmol/l) | 4.71 (3.88-5.66) | 4.53 (3.91-5.51) | 0.273 |
| Triglyceride (mmol/l) | 1.63 (1.07-2.47) | 1.42 (0.97-2.2) | 0.007 |
| Calcium (mmol/l) | 2.35 (2.3-2.43) | 2.35 (2.3-2.43) | 0.524 |
| Phosphate (mmol/l) | 1.2 (1.2-1.32) | 1.2 (1.07-1.32) | 0.546 |

AF, Animal Fluency test; CERAD-WL, Consortium to Establish a Registry for Alzheimer’s Disease Word Learning test; CERAD-DR, Consortium to Establish a Registry for Alzheimer’s Disease Delayed Recall test; DSST, Digit Symbol Substitution test; CKD, chronic kidney disease; BUN, blood urea nitrogen; eGFR, estimated glomerular filtration rate; UACR, urea albumin-creatinine ratio.
